# Protein-Inorganic Hybrid Nanoflowers as Efficient Biomimetic Antibiotics in the Treatment of Bacterial Infection

**DOI:** 10.3389/fchem.2021.681566

**Published:** 2021-04-30

**Authors:** Ying Zhou, Ying Li, Yunwei Fei, Mingrui Zhang, Shuang Wang, Fuqiu Li, Xingfu Bao

**Affiliations:** ^1^Department of Dermatology, Second Hospital of Jilin University, Jilin University, Changchun, China; ^2^Jilin Provincial Key Laboratory of Tooth Development and Bone Remodeling, Hospital of Stomatology, Jilin University, Changchun, China; ^3^Department of Cardiology, Second Hospital of Jilin University, Jilin University, Changchun, China

**Keywords:** reactive oxygen species, nanozymes, bacterial infections, antibacterial activity, biosafety

## Abstract

Nanozymes have been developed as new generation of biomimetic antibiotics against wound infection. However, most of new-developed nanozymes based on inorganic particles or hybrid ones usually originate from incompatible raw materials or unwanted metal salts, highly limiting their further biomedical usages. To overcome above drawbacks, it is highly required to develop novel nanozymes with great antibacterial activity by using biocompatible reagents and endogenous metal species as raw materials. Here, we demonstrated that bovine serum albumin enwrapped copper phosphate-based protein-inorganic hybrid nanoflowers possessed intrinsic peroxidase-like activity, which could be used as efficient biomimetic antibiotics against bacterial infection via the nanozyme-mediated generation of high toxic reactive oxygen species (ROS). With the admirable peroxidase-like activity, our nanoflowers could efficiently kill drug-resistance bacteria under physiological conditions, improve the wound healing after pathogen-induced infection, as well as avoid the potential tissue injury in time. Comprehensive toxicity exploration of these nanoflowers indicated their high biocompatibility and excellent biosafety. Our current strategy toward the design of protein-inorganic hybrid nanozymes with high biosafety and few side effects could provide a new paradigm for the development of nanozyme-based antibacterial platform in future.

## Introduction

As one of the greatest global health problems, bacterial infection has afflicted millions of people annually and attracted widespread attention in decades. Current treatments toward bacterial infection mainly rely on the use of various antibiotics, which lead to serious multidrug resistance and rapid decreases in therapeutic effect. To date, conclusive evidence indicates that antibacterial resistance is implicated in 700,000 deaths per year. More seriously, the number of deaths per year will increase to 10 million by 2050 unless essential action is taken (Grundmann et al., 2006; Chen et al., 2018). Taking together, recent clinical guidelines and fundamental research extremely demand effective means to block up the new emergence of multidrug-resistant bacteria.

As an accessible conventional antibacterial reagent, H_2_O_2_ is widely used in wound infusion and clinical treatment against inflammation. In general, H_2_O_2_ with a high concentrations is beneficial to antibacterial treatment, however can lead to adverse reaction toward healthy tissues (Wang et al., 2017, 2018a). In order to attack these shortcomings, development of novel antibacterial approaches becomes important on these challenges. Up to now, great efforts have been devoted to design novel antibacterial materials including organic molecules, cationic polymers, metal particles, organic-inorganic hybrid materials (Hindi et al., 2009; Huh and Kwon, 2011; Panacek et al., 2018; Peng et al., 2018; Li R. et al., 2020; Wang et al., 2020c). Among all these well-defined antibacterial materials, the burgeoning nanozymes have been considered as a new generation of biomimetic antibiotics to combat bacterial infection because of their high stability, long-term storage, broad-spectrum antibacterial activity, and absences of multi-drug resistance (Huang et al., 2016, 2019; Liang and Yan, 2019; Wu et al., 2019). For instance, a series of nanozymes including carbon-based materials, metal oxides, and transition metal single-atom nanocatalysts have been reported to possess intrinsic peroxidase-like activity, which can specifically catalyze the conversion of H_2_O_2_ into highly toxic reactive oxygen species (ROS) (Cai et al., 2015; Wang et al., 2018b, 2020a; Liu X. et al., 2019; Liu Y. et al., 2019). Therefore, nanozymes with peroxidase-like activity used together with H_2_O_2_ can overcome above-mentioned problems including unsatisfactory antibacterial effect under low concentration of H_2_O_2_ and unwanted side effects under high concentration of H_2_O_2_, exhibiting great potential in further treatment of wound infection. More importantly, extra characteristics of nanozymes, such as defect-rich adhesive, photodynamic, photothermal, and sonodynamic functionalities, can highly enhance their antibacterial activities both *in vitro* and *in vivo* (Cao et al., 2019; Li J. et al., 2019; Li S. et al., 2019; Liu Y. et al., 2019; Li Y. et al., 2020; Sun et al., 2020; Wang et al., 2020b). Although promising, the intrinsic components of currently reported nanozymes usually limit their further biomedical usages and clinical transformation. For example, carbon-based nanozymes prepared via solvothermal synthesis, chemical vapor deposition, oxidation stripping method, and oxidation reflux usually require special instruments and specific experimental conditions. Although exhibiting certain biosafety, these carbon-based nanozymes cannot metabolize or degrade in body (Zhang et al., 2012). Moreover, the raw materials toward the constructions of metal oxide-based nanozymes and single-atom nanozymes are highly dependent on the use of transition metal salts and organic solvents, as well as complicated synthesis conditions (Liu X. et al., 2019; Xu et al., 2019; Cao et al., 2020). Therefore, it is urgently needed to discover a new way to engineer nanozymes with admirable antibacterial activity and high therapeutic outcomes by using biocompatible reagents and endogenous metal species as raw materials.

In this study, we demonstrated that protein-inorganic hybrid nanoflowers with intrinsic peroxidase-like activity could act as efficient biomimetic antibiotics against bacterial infection and improve relative wound healing. These flower-like nanozymes composited with Cu_3_(PO_4_)_2_·3H_2_O nanocrystals and protein coating were prepared via a mild aqueous synthesis by using copper ions as inorganic components, biocompatible bovine serum albumin as nucleation template, as well as neutral phosphate buffer saline as reaction solvent. Our nanozyme-mediated antibacterial platform could efficiently kill drug-resistance bacteria under physiological conditions, improve the wound healing after pathogen-induced infection, and avoid the potential tissue injury in time. Moreover, these well-developed nanoflowers held extremely low long-term systemic toxicity, indicating their potentials in further biomedical usages and clinical transformation. Our current design could establish a new methodology for the construction of protein-inorganic hybrid nanozymes with high biosafety and few side effects, as well as provide a new direction into the optimization of novel nanozyme-based antibacterial platform.

## Chemicals and Methods

### Chemicals

Tetramethylbenzidine (TMB), 2, 2-azinobis (3-ethylbenzothiozoline)-6-sulfonic acid (ABTS), H_2_O_2_, and CuSO_4_ were achieved from Aladdin Reagent. Calcein AM, propidium iodide (PI), fluorescein diacetate (FDA), dimethyl sulfoxide (DMSO), and bovine serum albumin (BSA) was purchased from Sigma-Aldrich. All other reagents were of analytical grade and used directly without further purification. Ultrapure water was used throughout all the experiments.

### Measurements

Scanning electron microscopy (SEM) images were obtained on a field-emission SEM instrument (S-4800). Transmission electron microscopy (TEM) images were obtained using a high-resolution transmission electron microscope (FEI TECNAI G2 F20). X-ray diffraction (XRD) analysis was performed on a Focus diffractometer (Bruker-D8). Ultraviolet-visible (UV-vis) spectroscopy was determined via a UV-vis spectrometer (JASCO V-550). Fluorescence microscopy images of cells and bacteria were collected on an Olympus imaging system (BX-51).

### Synthesis of BSA-Cu_3_(PO_4_)_2_·3H_2_O Nanoflowers

CuSO_4_ solution (120 mM, 1 mL) was added to PBS (0.1 M, pH 7.4, 150 mL) containing bovine serum albumin (0.5 mg/mL). Then, the mixture was incubated at room temperature for 3 days. Subsequently, as-prepared nanoflowers were isolated via centrifugation, washed with ultrapure water, and freeze-dried overnight.

### Peroxidase-Like Activity Measurements

The peroxidase-like activity of nanoflowers were investigated by using the catalytic oxidation of peroxidase substrate TMB or ABTS in the presence of H_2_O_2_, respectively. To examine the activity of nanoflowers (NFs) as a catalyst on the oxidation of TMB, four groups were defined as control, NFs, H_2_O_2_, and NFs+H_2_O_2_. Concentrations of TMB, NFs, and H_2_O_2_ used in the typical experiments were defined as 1 mM, 20 μg/mL, and 25 mM, respectively. Experiments were carried out in phosphate-buffered saline (PBS) (pH 4.0, 10 mM) and monitored at 652 nm on a UV-vis spectrometer. For the oxidation of ABTS, concentrations of ABTS, NFs, and H_2_O_2_ in the typical experiments were 1 mM, 20 μg/mL, and 25 mM, respectively. Experiments were performed in PBS (pH 4.0, 10 mM) and monitored spectrophotometrically at 417 nm. To determine the effects of pH value and temperature on the catalytic activities of nanoflowers, PBS (10 mM) with different pH values and PBS (pH 4.0, 10 mM) with different reaction temperatures were selected and used in current study, respectively.

### Cell Cultures

Human umbilical vein endothelial cells (HUVEC) were supplied by ATCC (American Type Culture Collection). Cells were cultured in Dulbecco's modified Eagle's medium (DMEM) containing 10% fetal bovine serum (FBS) in an incubator (37°C, 5% CO_2_). Cells were harvested by the use of trypsin and were re-suspended in fresh medium before plating.

### Cytotoxicity

Cells were cultured in a 96-well plate for 12 h to allow the cellular attachment. Then, nanoflowers with different concentrations were added into above medium. 12 h later, medium containing nanoflowers was removed, and cells were treated with MTT for another 4 h. DMSO was used to dissolve the formazan crystals, and the absorbance at a wavelength of 570 nm was determined on a microplate reader (*n* = 4). Cell viabilities were normalized to the viability of cells without any treatment.

### Cellular Viability Observation

For cellular viability observation, cells were plated in a 6-well plate for 12 h to allow the cellular attachment. Then, nanoflowers with different concentrations were co-incubated with cells for another 12 h. After medium containing nanoflowers was removed, live-dead staining was carried out and stained cells were observed under a fluorescence microscopy.

### Animal Administration

Female ICR mice (25 g, 6 weeks) were purchased from Laboratory Animal Center of Jilin University. Protocol of all the animal studies was approved by the Institutional Animal Care and Use Committee at Jilin University.

### Analysis of Hemolysis and Coagulation

Fresh mouse blood samples stabilized by heparin were obtained from anesthetic ICR mice. After centrifugation, red blood cells (RBCs) were diluted to 1/4 of their volumes with 0.9% NaCl solution. Diluted RBCs (0.2 mL) were mixed with 0.9% NaCl solution (0.8 mL) as the negative group, water (0.8 mL) as the positive group, and 0.9% NaCl solution containing nanoflowers with different concentrations (0.8 mL) as experimental samples. All the samples were vortexed and kept at room temperature for 3 h. Then, the absorbance of supernatants at 541 nm was determined on a UV-vis spectroscopy (*n* = 4). Hemolysis rates = (sample absorbance-negative absorbance)/(positive absorbance-negative absorbance). For the coagulation assays, plasma was mixed with 0.9% NaCl solution containing nanoflowers with different concentrations at first. Then, a fully automatic blood coagulation analyzer was used to confirm the values of APTT and PT (*n* = 4).

### Bacterial Culture and Antibacterial Experiments

Monocolony of drug-resistant *Staphylococcus aureus* (*S. aureus* ATCC 43300) was firstly transferred to a liquid Luria-Bertani (LB) broth and shaken at 37°C for 12 h. Then, bacteria were harvested and diluted to 10^6^ CFU/mL with LB broth. In the typical experiment, bacteria and H_2_O_2_ with different concentrations were incubated in LB culture medium at 37°C in the presence or absence of nanoflowers (100 μg/mL). 6 h later, bacterial concentrations were detected by measuring the optical density at 600 nm (OD_600_). OD_600_ of LB broth containing bacteria was defined as control while OD_600_ of LB broth was defined as background (*n* = 4). Survival rates = (OD_600_ of test groups – OD_600_ of background)/(OD_600_ of control – OD_600_ of background) × 100%. For the spread plate method, bacteria after different treatments were harvested and diluted with LB broth. Solutions containing bacteria (100 μL) were spread on solid LB agar plates and cultured at 37°C for 6 h. For live/dead staining analysis, FDA and PI were mixed with bacteria after different treatments in dark for 30 min. Then, achieved mixtures were washed with 0.9% NaCl solution and observed under fluorescence microscopy. The concentrations of H_2_O_2_ and nanoflowers in above spread plate experiment and live-dead staining were 25 mM and 100 μg/mL, respectively. For SEM images, *S. aureus* after various treatments were harvested, washed with 0.9% NaCl solution, and fixed in 4% formaldehyde. 4 h later, *S. aureus* were rinsed with 0.9% NaCl solution again and dehydrated using ethanol. Then, *S. aureus* were dried in dark at room temperature. In addition, monocolony of drug-resistant *Escherichia coli* (*E. coli* ATCC 25922) was used to re-confirm the antibacterial activity of our well-prepared nanoflowers.

### Establishment of Bacteria-Infected Wounds and Antibacterial Treatment

To explore the efficacy of nanoflowers in the treatment of bacteria-infected wounds *in vivo*, bacteria-infected wounds on mice were established at first. Round wounds with an average diameter of 5 mm were created on the back of mice at first. Then, drug-resistant *S. aureus* were added on above wounds. After being infected, mice were randomly divided into four groups, which were defined as control (0.9% NaCl solution), NFs, H_2_O_2_, and NFs+H_2_O_2_, respectively (*n* = 4). To investigate the effects of antibacterial treatments, wounds were dressed with medical Band-Aids with or without nanoflowers. The dosage of H_2_O_2_ used in this study was 0.25 μmol per wound (10 mM, 25 μL) while the dosage of nanoflowers was 10 μg per wound (100 μg/mL, 100 μL). Band-Aids were changed every day and H_2_O_2_ was added at the same time. The whole therapeutic process was defined as 4 days, and photos of wounds were taken from above groups at each expected time points.

### Skin Sensitivity and Long-Term Toxicity Investigation of Nanoflowers

ICR mice without hair on their back were randomly divided into two groups, which were defined as control group (none treatment) and test group (skin exposure toward Band-Aids containing nanoflowers), respectively (*n* = 4). Band-Aids containing nanoflowers (20 μg per Band-Aid) were changed 3-day interval. Mouse body weights were recorded for 30 days. 30 days after first grouping, mouse blood was collected to perform biochemical assay. After mice were sacrificed, main organs were harvested, fixed in buffered formalin, embedded in paraffin, sectioned, and stained with hematoxylin-eosin (H&E).

## Results and Discussion

**Figure 1:**
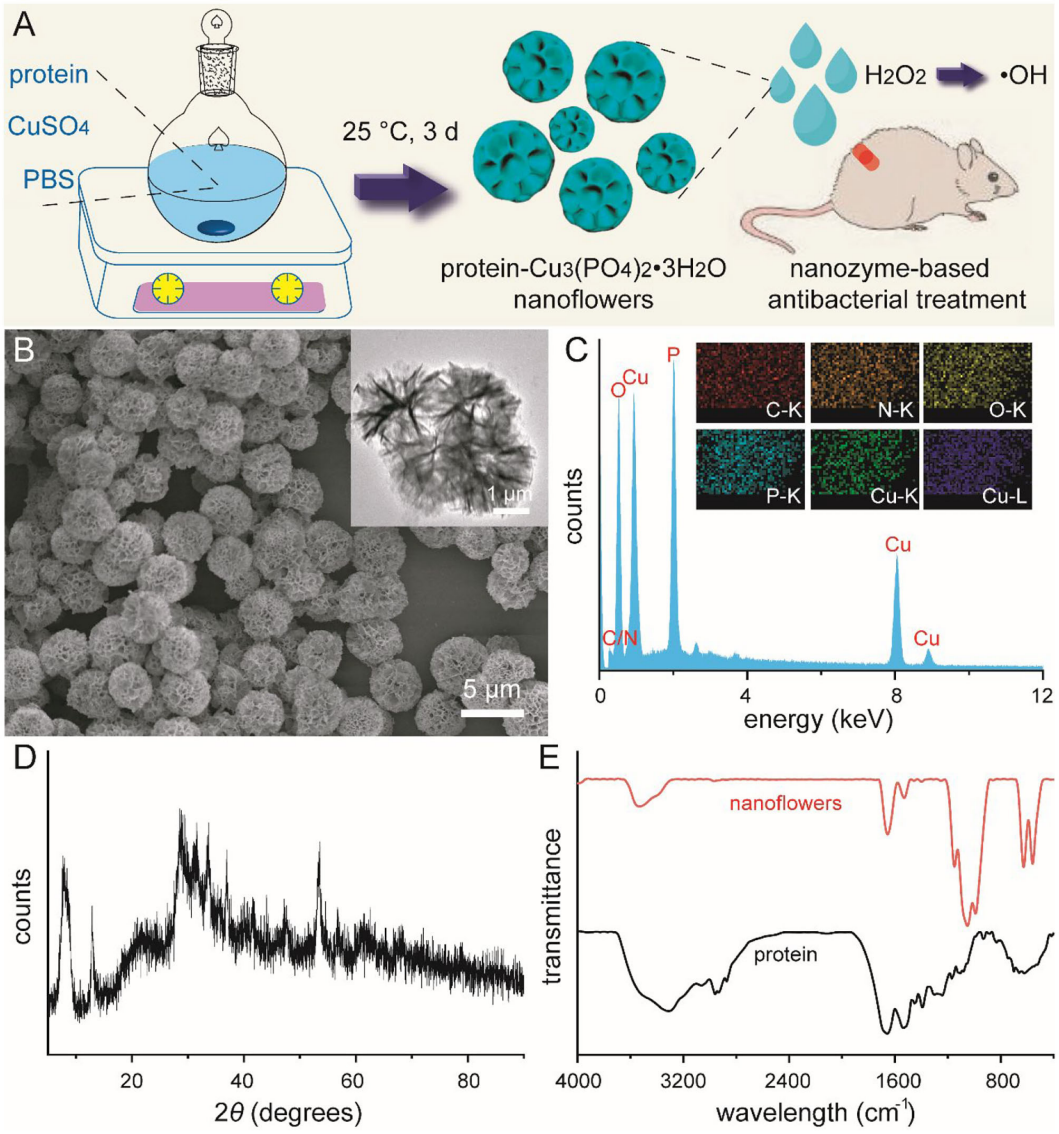
Schematic illustration for the mild synthesis of protein-Cu_3_(PO_4_)_2_·3H_2_O nanoflowers and their usage in nanozyme-based antibacterial treatment **(A)**. SEM image **(B)**, EDS analysis **(C)**, and wide-angle XRD pattern **(D)** of nanoflowers. Inset of **(B)**: TEM image of nanoflowers. Inset of **(C)**: elemental mapping of nanoflowers. FT-IR spectrum of bovine serum albumin and nanoflowers **(E)**.

Mild synthesis of protein-Cu_3_(PO_4_)_2_·3H_2_O nanoflowers and their potential usage in nanozyme-based antibacterial treatment was schematically illustrated in Figure 1A (Ge et al., 2012; Huang et al., 2015). In our current design, bovine serum albumin (BSA) was selected as the template protein in the typical synthesis according to its high biocompatibility, easy accessibility, and inexpensive characteristic. Copper ions with relatively low concentration were selected and used as metal components, which were reported to improve wound healing via stimulating cell migration and angiogenesis (Gopal et al., 2014; Kornblatt et al., 2016; Qiu et al., 2020). During the mild aqueous synthesis with a period of 3 days, these well-designed nanoflowers were gradually formed as blue precipitates. As shown in Figure 1B, typical images from scanning electron microscopy (SEM) and transmission electron microscopy (TEM) indicated that these newly-developed protein-inorganic hybrid materials held a monodispersed flower-like morphology. When the volume of aqueous solution and concentration of BSA were defined as 150 mL and 0.5 mg/mL, these nanoflowers revealed an average diameter of 5.5 μm with a relatively narrow particle size distribution. Moreover, branch-like microstructure could be well-detected around the edge of nanoflowers based on TEM image. Chemical composition of these nanoflowers was then confirmed by energy-dispersive spectroscopy (EDS) pattern and corresponding elemental mappings. Figure 1C and relative inset indicated the presence and uniform distribution of C, N, O, P, and Cu in these nanoflowers. Wide-angel X-ray diffraction (XRD) pattern of these blue nanoflower powder shown in Figure 1D fitted that of Cu_3_(PO_4_)_2_·3H_2_O (00-022-0548), further indicating that the basic ingredients of these nanoflowers were inorganic Cu_3_(PO_4_)_2_·3H_2_O and amorphous BSA components. Fourier transform infrared (FT-IR) spectrography was then used to confirm the chemical structure of these nanoflowers (Figure 1E). Absorption band between 1,400 and 1,600 cm^−1^ could be detected in both BSA and nanoflowers owing to the multiple characteristic asymmetric and symmetric of carboxyl group. In addition, broad peaks around 3,200 cm^−1^ could be corresponded to the presence of H_2_O molecules and hydrogen bonding, which demonstrated that protein used in the current system could well lead to the nucleation of these hybrid materials. All these results indicated the successful synthesis of protein-inorganic hybrid nanoflowers.

**Figure 2:**
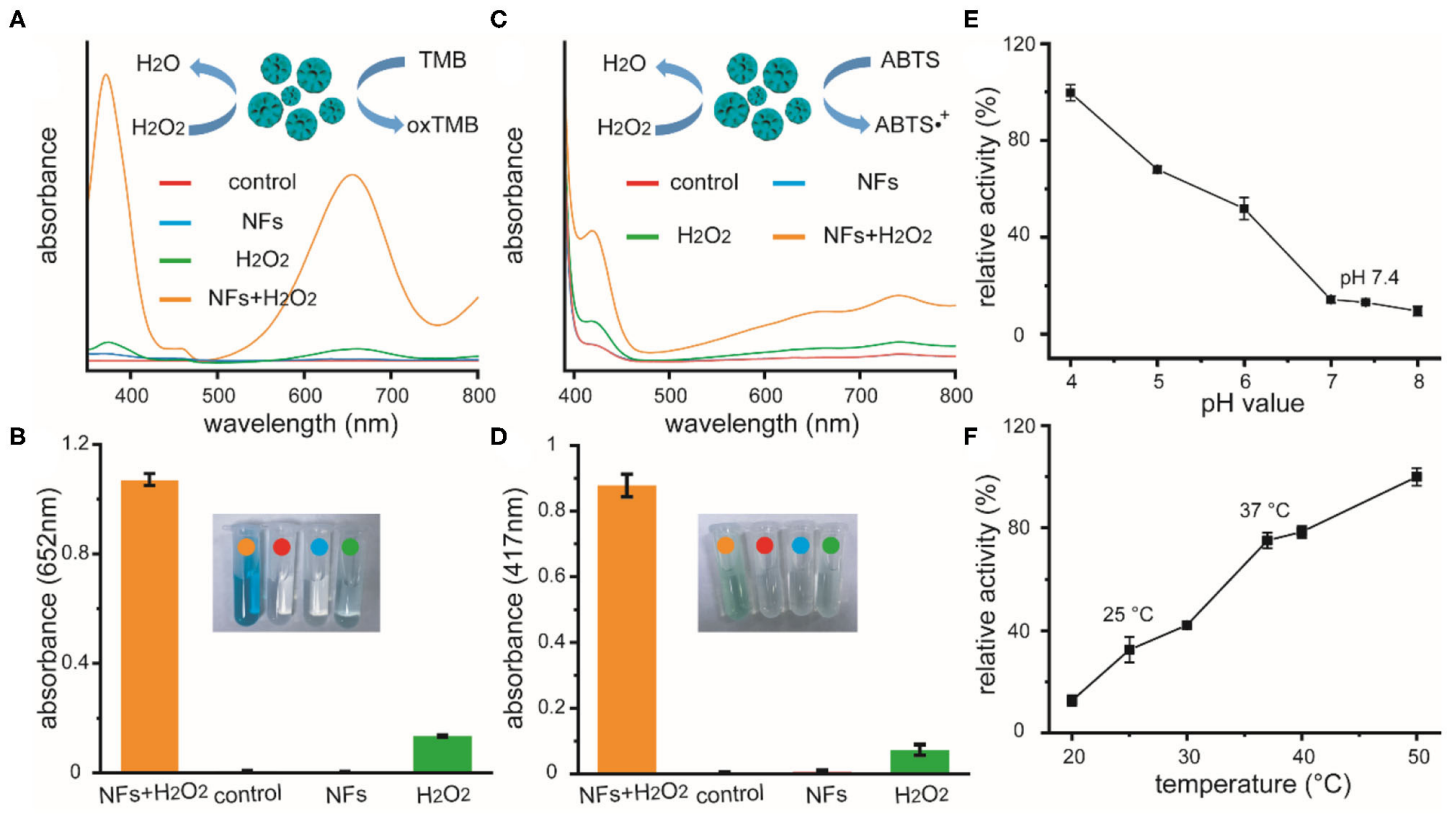
Absorption curves of TMB solutions **(A)** and ABTS solutions **(C)** upon different treatments, as well as relative quantitive absorbance changes at 652 or 417 nm as a result of the catalyzed oxidation of TMB **(B)** or ABTS **(D)**, respectively. Inset of **(A,C)**: schematic illustration for the peroxidase-like catalytic activity of nanoflowers in the presence of TMB or ABTS. Inset of **(B,D)**: photographic images of corresponding solutions after different treatments. Effects of pH value **(E)** and temperature **(F)** on the catalytic activities of nanoflowers.

After the formation and characterization of these nanoflowers, we further explored the peroxidase-like activity of these new-developed materials. The catalytic oxidation of peroxidase substrate TMB and ABTS were spectroscopically evaluated, respectively. After co-incubating H_2_O_2_ with these nanoflowers and TMB for 10 min in acid PBS at 37°C, the color of solution turned blue with a strong absorption at 625 nm. Results based on other experimental groups revealed that solutions containing nanoflowers or TMB could not lead to any color change, indicating the presence of peroxidase-like catalytic activity of our well-prepared nanoflowers. Besides, H_2_O_2_ could induce slight color change, which could be ascribed to homolytic cleavage of H_2_O_2_. Possible catalytic mechanism based on TMB system of our catalyst was summarized in the inset of Figure 2A. Quantitive experiments for catalytic activity of nanoflowers (20 μg/mL) in the presence of H_2_O_2_ (25 mM) were performed at least 3 times. Relative results and photo of color change were provided in Figure 2B. Besides, the peroxidase-like catalytic activity of our nanoflowers was also evaluated by oxidizing chromogenic substrate ABTS upon the assistance of H_2_O_2_ (Figures 2C,D). Our current studies exhibited similar results with some previous researches focused on the investigation of catalytic activity for copper-based micro-nano materials. It was well-known that catalytic activity of nanozymes was highly dependent on the relative reaction conditions, therefore the effects of pH value and temperature on catalytic activity were explored in detail, respectively. In detail, TMB molecules were selected and used as chromogenic substrate in above investigations. As shown in Figure 2E, the catalytic activity of our nanoflowers decreased gradually in the pH values range from 4 to 8, demonstrating that weak acidity was the optimal reaction condition. However, nearly 20% catalytic activity of these nanoflowers could be well-retained under neutral condition, indicating further potential biomedical applications of these nanoflowers. Moreover, our nanoflowers with high-temperature resistance exhibited high catalytic activity even at 50°C, and the catalytic activity increased dramatically with the increasing of temperature (Figure 2F). All these results demonstrated that our nanoflowers with wide pH range applicability and great thermal stability could be widely used in various nanozyme-based catalytic theranostics.

**Figure 3:**
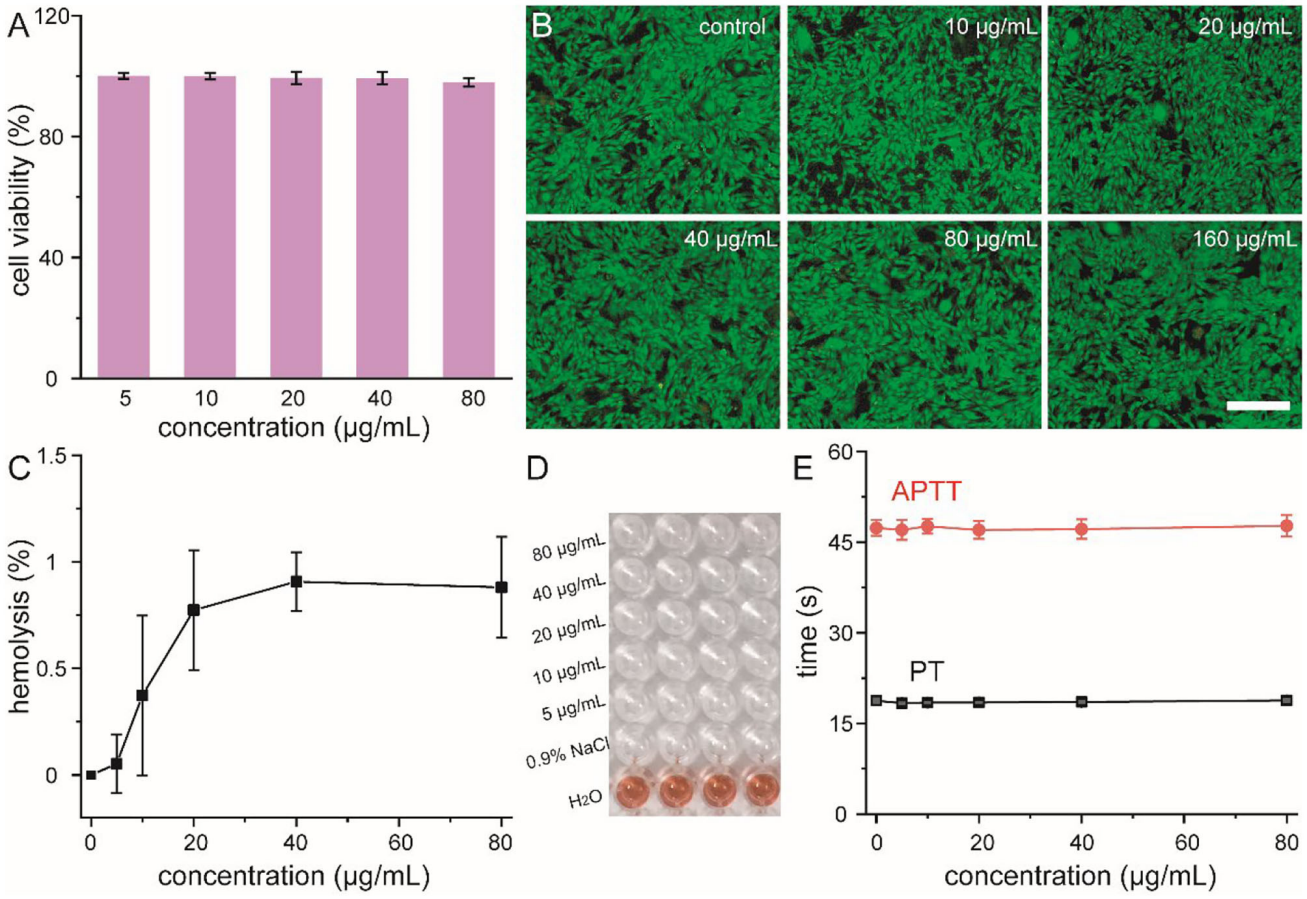
Cell viability **(A)** and live-dead staining **(B)** of HUVEC cells treated with nanoflowers with different concentrations. Scale bar was equal to 100 μm. Hemolysis percentages **(C)** of nanoflowers and relative photographic image **(D)**. Nanoflower-induced blood coagulation analysis **(E)**.

Prior to the use of our nanoflowers as biomimetic antibiotics in the treatment of bacterial infection both *in vitro* and *in vivo*, their cytotoxicity and blood compatibility were explored in detail (Bao et al., 2018). Methyl thiazolyl tetrazolium (MTT) assay was performed to determine the relative viabilities of HUVEC toward nanoflowers at first. As shown in Figure 3A, all the cell viabilities were not hindered after co-incubation with different concentrations of nanoflowers, indicating that these nanoflowers exhibited negligible cytotoxicity. Moreover, results of live-dead staining revealed that dead staining could not be found based on the fluorescence images, indicating the high biocompatibility of these nanoflowers (Figure 3B). Hemolytic assay was then used to investigate the interaction between nanoflowers and blood components. As shown in Figures 3C,D, all the hemolysis percentages were lower than 1% based on both qualitative and quantitative results, indicating these nanoflowers were not even able to induce slight injury of red blood cells. As two essential factors to assess the extrinsic and intrinsic coagulation routes, prothrombin time (PT) and activated partial thromboplastin time (APTT) were selected to explore the blood coagulation of these nanoflowers. No obvious differences could be detected between the nanoflower-treated groups and control, therefore demonstrating the effect of nanoflowers on the blood coagulation time could be negligible (Figure 3E). All of these essential exploration validated the overall safety of nanoflowers *in vitro*.

**Figure 4:**
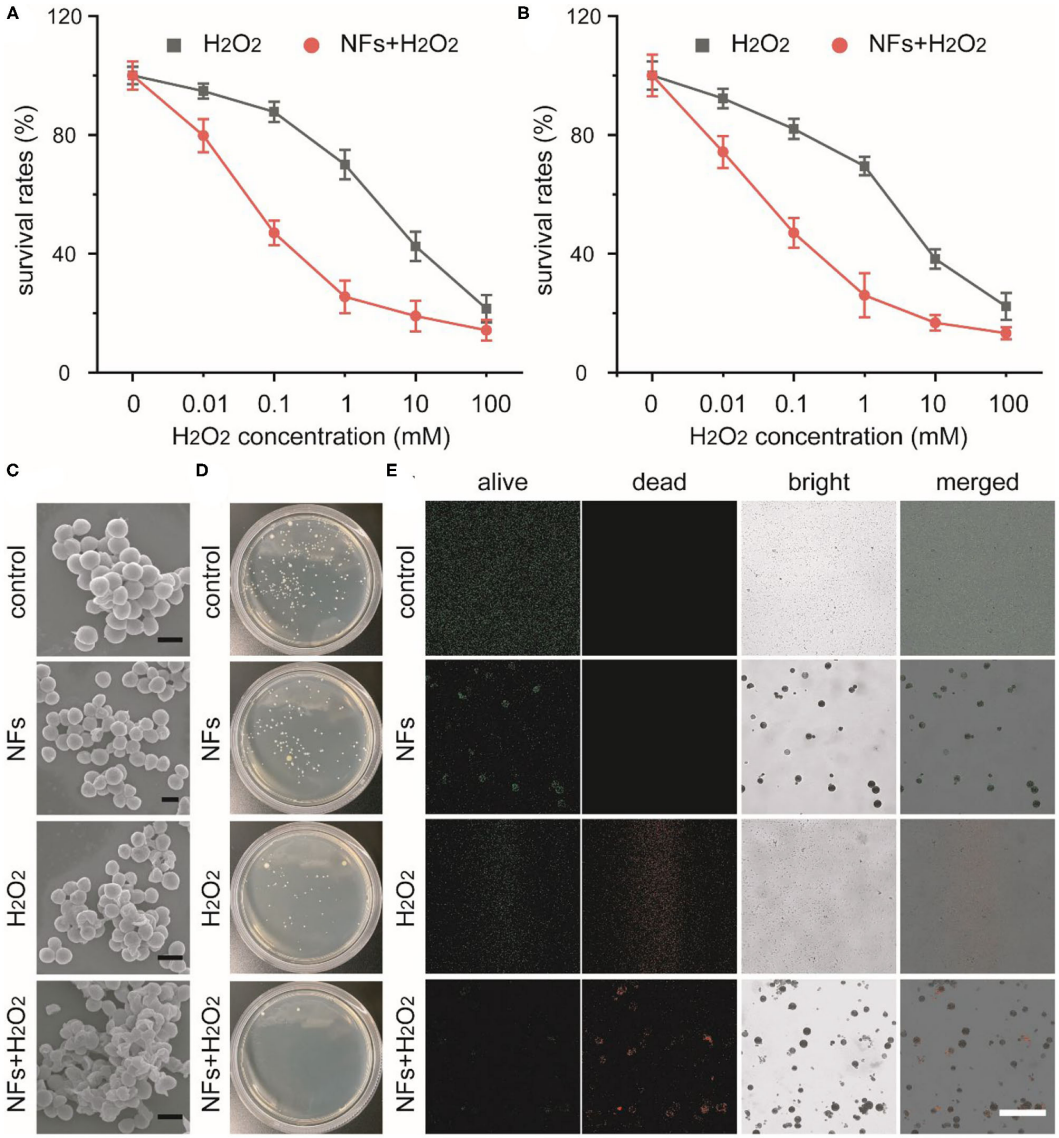
Concentration-dependent survival percentages of *S. aureus* **(A)** and *E. coli* **(B)** treated with H_2_O_2_ in presence or absence of nanoflowers. SEM images of *S. aureus* after various treatment **(C)**. Scale bars was equal to 1 μm. Photos of bacterial colonies formed by *S. aureus* after different treatments **(D)**. Live-dead staining of *S. aureus* after different treatments **(E)**. Scale bar was equal to 50 μm.

Recent studies indicated that nanozymes with peroxidase-like activity could catalyze H_2_O_2_ into highly toxic ·OH and avoid unwanted tissue toxicity and injury around wounds. Our current research indicated that these well-prepared nanoflowers exhibited great peroxidase-like catalytic activity under pathological conditions. Taking together, we develop a nanozyme-mediated antibacterial platform against drug-resistant bacteria to explore the feasibility of our nanoflowers as efficient peroxidase mimics to enhance the generation of reactive oxygen species (ROS). In our experimental design, drug-resistance *S. aureus* and *E. coli* were selected as typical bacteria to explore the antibacterial ability of our nanozyme-based platform. As shown in Figures 4A,B, H_2_O_2_ with low concentrations could not exhibited obvious antibacterial activity. However, with the addition of our nanoflowers, bacterial viabilities decreased significantly. Compared with H_2_O_2_ alone, above results implied that our nanoflowers with intrinsic peroxidase-like activity could enhance the antibacterial ability in the presence of H_2_O_2_ with the same concentrations through the efficient nanozyme-based catalytic process and the generation of high toxic ROS. SEM images shown in Figure 4C indicated the changes of *S. aureus* morphology affected by our current system. No damages could be found in the group of nanoflowers as compared with the group of control. However, bacterial membrane exhibited slightly rough and wrinkled in the group of H_2_O_2_ while bacterial surface was seriously damaged by the highly toxic ·OH. Luria-Bertani agar plate assay was then used to confirm the viable counts of bacteria from different groups (Figure 4D). As expected, colony-forming in the group of NFs+H_2_O_2_ was nearly totally inhibited as compared with that in the group of control. A mass of colonies could be detected in the groups of NFs, indicating that our nanoflowers did not held antibacterial effect. The numbers of colonies in the group of H_2_O_2_ was between those in groups of control and NFs+H_2_O_2_, demonstrating H_2_O_2_ alone with the same concentration could exhibit slight antibacterial effect. Visible antibacterial performance of our nanoflowers was further confirmed by live (green)-dead (red) staining analysis using fluorescence imaging (Figure 4E). Red fluorescence signal could be easily detected in the group of NFs+H_2_O_2_ while nearly no green signal could be found around our nanoflowers in the merged image, indicating that nearly all the bacteria were killed upon the nanozyme-based antibacterial treatment. However, only green fluorescence signal was found in the group of NFs, additionally implying that our nanoflowers did not held antibacterial effect. Both red fluorescence signal and green signal could be detected in the group of H_2_O_2_, indicating H_2_O_2_ held some antibacterial effect. Significantly, higher red fluorescence signal could be found in the group of NFs+H_2_O_2_ compared to that in the group of H_2_O_2_, strongly validating the excellent bacterial killing efficiency of our nanoflowers in the presence of H_2_O_2_. These aforementioned results proved the high-performance antibacterial effect of our well-developed nanozyme-mediated platform.

**Figure 5:**
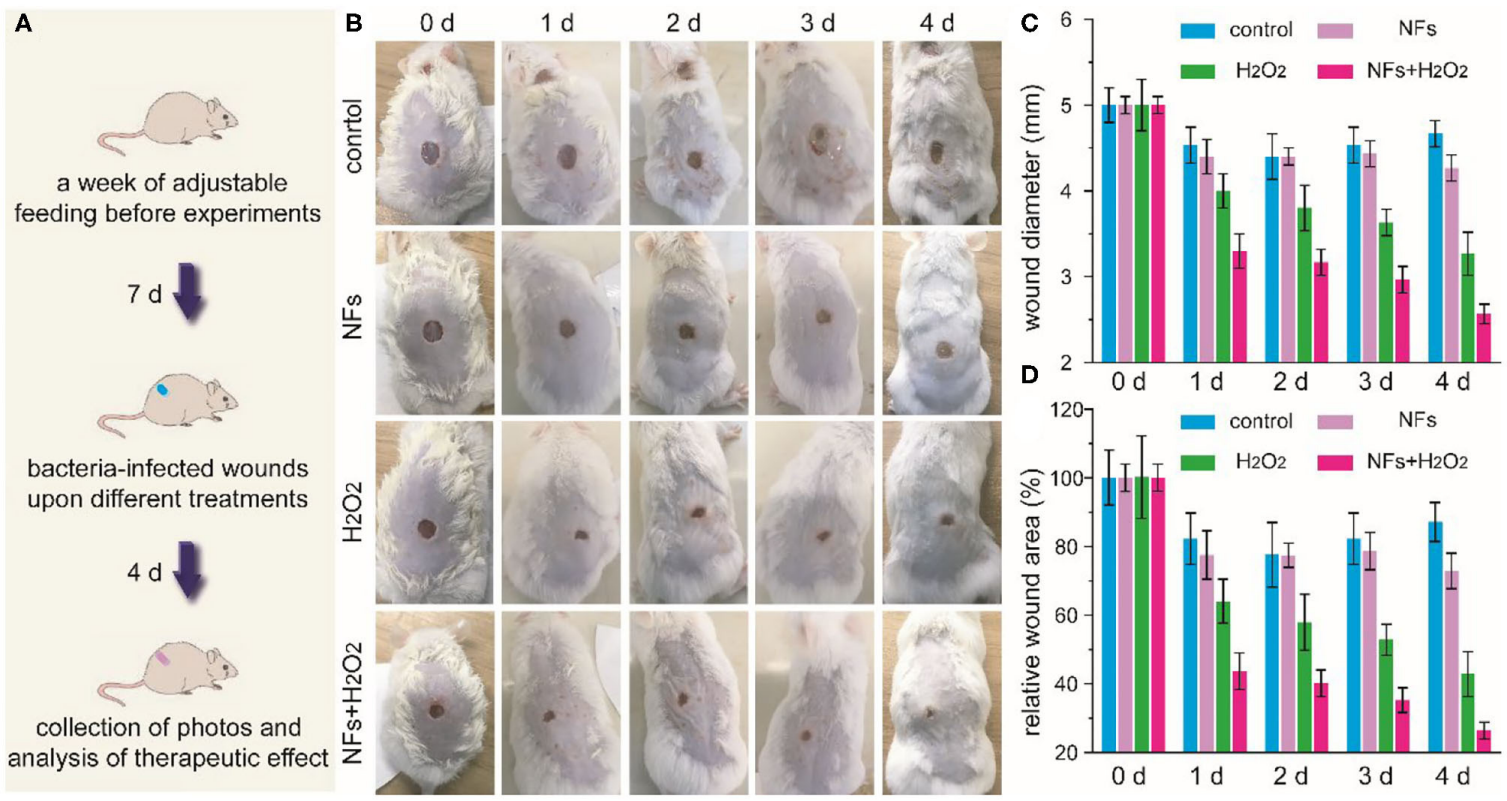
Schematic illustration of drug-resistance *S. aureus*-induced infection and the following experimental design of antibacterial treatment **(A)**. Time-dependent photos of bacteria-infected wounds **(B)**, wound diameters **(C)**, as well as relative wound areas **(D)** after different treatments during the therapeutic process.

Encouraged by the outstanding antibacterial effect of current nanozyme-mediated platform, we further explored the feasibility of these nanoflower-based biomimetic antibiotics to treat bacteria-infected wounds *in vivo*. Drug-resistance *S. aureus*-induced infection and relative design of nanozyme-mediated antibacterial treatment were schematically illustrated in Figure 5A. Firstly, round wounds with a diameter of 5 mm were created on the back of mice and drug-resistant *S. aureus* were dropped on above wounds to create bacteria-infected model. Secondly, mice after being infected were randomly divided into 4 groups including control, NFs, H_2_O_2_, and NFs+H_2_O_2_. Thirdly, wounds were dressed with medical Band-Aids with or without nanoflowers in the presence of absence of H_2_O_2_, respectively. As shown in Figure 5B, wounds of mice in the groups of control and NFs exhibited obvious edema and inflammation while wounds of mice in the group of NFs appeared less inflammation in the whole therapy process. Quantified changes of wound diameter and relative area were summarized in Figures 5C,D. As compared with other groups, wounds in the group of NFs+H_2_O_2_ decreased significantly after the antibacterial treatment. Moreover, the group of NFs+H_2_O_2_ held the best wound closure effect among all the groups. These exciting results indicated the outstanding antibacterial activity of nanozyme-mediated platform *in vivo* and its extreme enhancement in wound healing.

**Figure 6:**
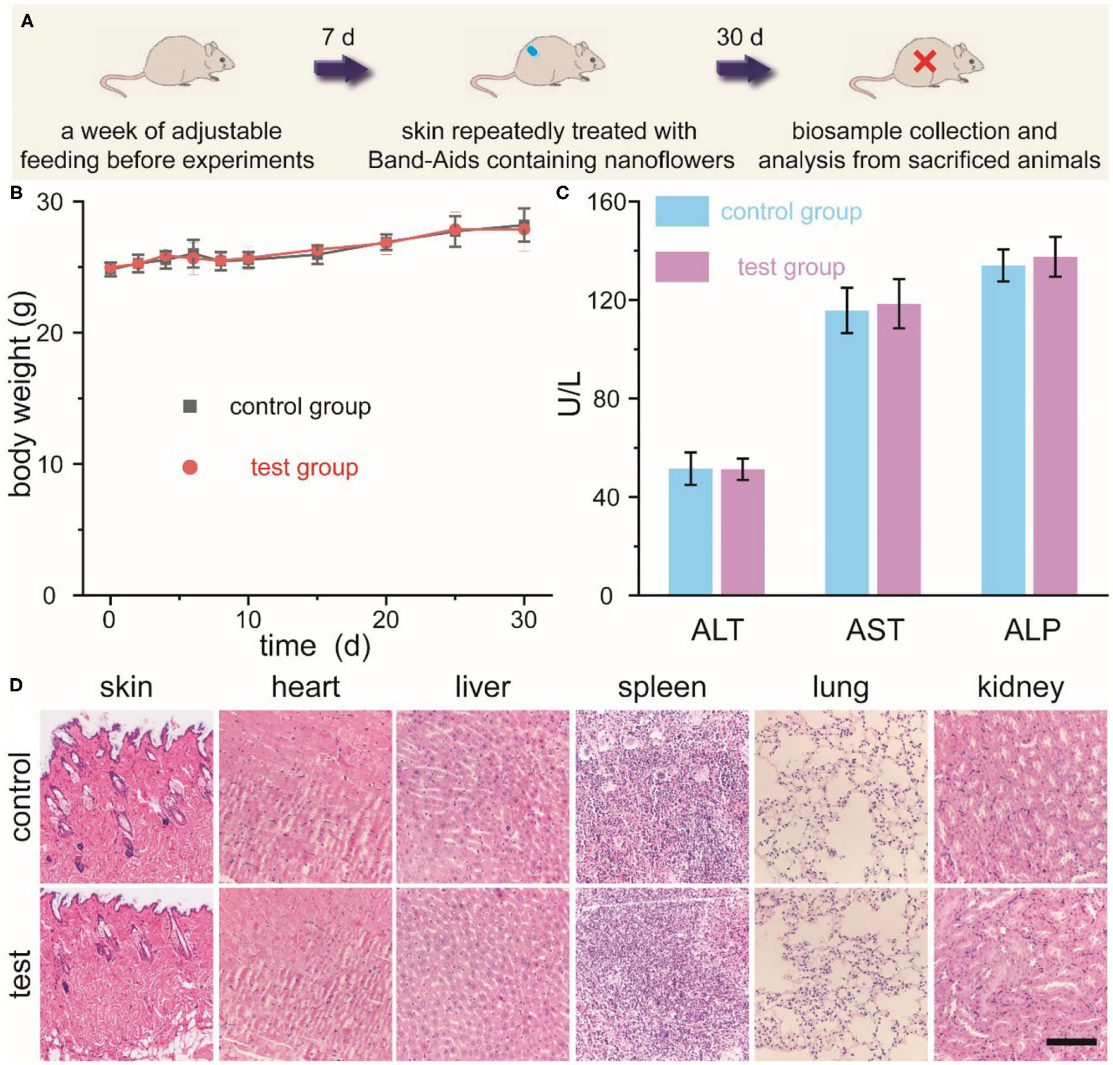
Schematic illustration for the experimental design of skin sensitivity and long-term toxicity investigation of nanoflowers **(A)**. Changes in body weight **(B)**, main blood biochemical assay **(C)**, as well as histological observation **(D)** of mice repeatedly treated with or without Band-Aids containing nanoflowers. Scale bar was equal to 200 μm.

Last but not least, we further investigated the skin sensitivity and long-term toxicity of these newly-developed nanoflowers in detail. Schematic illustration of our current experimental design was shown in Figure 6A. Firstly, mice without hair on their back were randomly divided into two groups, which were defined as the control group and the test group. Secondly, Band-Aids containing nanoflowers were dressed on the skin and changed 3-day interval. Thirdly, qualitative and quantitative biosafety indicators were then collected and anlysized. As the most direct method to evaluate the toxicity of any newly-developed materials, animal behavior and body weight were selected and assess in this study. As shown in Figure 6B, no obvious differences in the body weight were found between the control group and the test group. Moreover, no differences in eating, drinking, and behavior could be detected at the same time. To achieve more quantitative evaluation of these nanoflowers, blood biochemical analysis after different treatment was introduced. Figure 6C revealed that well-developed nanoflowers did not alter the hepatic functions of ICR mice including alanine aminotransferase (ALT), alkaline phosphatasein (ALP), and aspartate aminotransferase (AST). In addition, ematoxylin and eosin (H&E) histology analysis of directly contacted skin and main organs 30 days after the first administration of nanoflowers demonstrated that there were no obvious signs of inflammation and injuries occurred in the test group as compared with the control group (Figure 6D). Thus, all of above results indicated that our well-designed nanoflowers at the current given dosage exhibited high biosafety and low systemic toxicity.

## Conclusion

In summary, we rationally designed and prepared protein-inorganic hybrid nanoflowers with intrinsic peroxidase-like activity, which could be used as efficient biomimetic antibiotics against bacterial infection through the nanozyme-mediated generation of high toxic reactive oxygen species. In the typical synthesis, these well-defined nanoflowers were developed via a simple and mild aqueous synthesis in merge of biocompatible bovine serum albumin as nucleation template and neutral phosphate buffer saline as reaction solvent. These flower-like nanozymes could catalyze low level of H_2_O_2_ into highly toxic reactive oxygen species, which were verified by using TMB and ABTS as different peroxidase substrates. Moreover, spectroscopic results indicated that these flower-like nanozymes held wide pH range applicability and great thermal stability. Results of both *in vitro* and *in vivo* antibacterial experiments demonstrated that these nanoflowers with the admirable peroxidase-like activity could efficiently kill drug-resistance bacteria under physiological conditions. More importantly, our current nanozyme-mediated antibacterial platform could improve the wound healing after pathogen-induced infection, and avoid the potential tissue injury in time. Last but not least, these well-developed nanoflowers exhibited high biocompatibility and low long-term systemic toxicity, indicating their potential in further biomedical applications. We hoped that our study not only established a new strategy toward the synthesis of efficient protein-inorganic hybrid nanozymes with highly biocompatible raw materials, but also achieve the optimization of novel nanozyme-based antibacterial platform for further clinical transformation.

## Data Availability Statement

The raw data supporting the conclusions of this article will be made available by the authors, without undue reservation.

## Ethics Statement

The animal study was reviewed and approved by the Institutional Animal Care and Use Committee at Jilin University.

## Author Contributions

YZ, FL, and XB conceived and designed the experiments and wrote the manuscript. YZ, YL, YF, MZ, and SW performed the experiments. All authors analyzed the data and concluded the research.

## Conflict of Interest

The authors declare that the research was conducted in the absence of any commercial or financial relationships that could be construed as a potential conflict of interest.
